# ONLINE ASYNCHRONOUS INTERVENTION FOR FAMILY CAREGIVERS OF PERSONS WITH LEWY BODY DEMENTIA (LBD)

**DOI:** 10.1093/geroni/igae098.1080

**Published:** 2024-12-31

**Authors:** Oleg Zaslavsky, Jasmine Kaneshiro, Yan Su, Kimiko Domoto-Reilly, Annie T Chen

**Affiliations:** University of Washington, Seattle, Washington, United States; University of Washington, Seattle, Washington, United States; University of Massachusetts Dartmouth, Dartmouth, Massachusetts, United States; University of Washington, Seattle, Washington, United States; University of Washington, Seattle, Washington, United States

## Abstract

Family caregivers of individuals with Lewy Body Dementia (LBD) experience higher levels of stress and more severe depressive symptoms compared to caregivers of people with other dementias. This pilot study evaluated the efficacy of VOCALE LBD, an online asynchronous 8-week intervention aimed at improving psychological distress among LBD caregivers. We conducted a quasi-experimental study with 39 family caregivers. The program employs a web-based platform and includes text-based peer support, moderation of the discussion by a research team member, didactic training, and problem-solving skills development, all tailored to LBD caregiving situations. We assessed outcomes including caregiver depression, burden, stress, and support, at baseline, post-intervention (eight weeks), and one-month follow-up. We also evaluated problem solving, self-efficacy and mastery mechanistic variables. Participants’ average age was 68 (SD=10), with 69% female, 95% identifying as White, and 79% holding undergraduate and graduate degrees. The eight-week intervention had 18% attrition rate. Effect sizes (Cohen’s d) from baseline to post-intervention and from baseline to follow-up showed reductions in depression (-0.36 and -0.54, respectively), stress (-0.30 and -0.45), burden (-0.31 and -0.26), and increases in support (0.44 and 0.46). Significant improvements were observed in problem-solving skills, though no significant changes were noted in mastery and self-efficacy. The study confirms the acceptability and efficacy of the VOCALE LBD program to reduce psychological distress among family LBD caregivers and suggests activation of the targeted change mechanisms. The study also contributes to the field’s understanding of online support mechanisms and promotes the exploration of innovative caregiver support strategies.

